# Surgicel-Induced Anaphylaxis Post Permacath Placement

**DOI:** 10.7759/cureus.16938

**Published:** 2021-08-06

**Authors:** Michael C Corti, Anish D Thomas, Mark N Sayegh, Kasun Vernon, Cara Sherman, Robert Trainor

**Affiliations:** 1 Internal Medicine, St. Johns Riverside Hospital, Yonkers, USA; 2 Internal Medicine, Lake Erie College of Osteopathic Medicine, Erie, USA

**Keywords:** surgicel, anaphylaxis, post operative, permacath placement, allergic reaction, angioedema, hypersensitivity, cellulose

## Abstract

This case report details the resulting anaphylaxis and angioedema following placement of Surgicel hemostatic agent in a 38-year-old male postoperatively. Our patient experienced minor postoperative bleeding at the placement site of a dialysis catheter, which was controlled using Surgicel. Within minutes of the placement of Surgicel in the incision, the patient experienced an anaphylactic reaction with facial angioedema resulting in a Rapid Response being called to intervene. Incidences of Surgicel-induced anaphylaxis and hypersensitivity reactions are rare, but this report aims to bring awareness to this potential complication, as well as to assist with guiding management of future adverse reactions and surveillance of patients afterward.

## Introduction

Surgicel absorbable hemostat is an absorbable knitted fabric made of oxidized regenerated cellulose. Surgicel is used to accelerate clotting during and after surgeries, but the mechanism by which it does so is not entirely clear. Adverse events related to the placement of Surgicel are rare with only a few documented cases of an allergic reaction following its placement. Anaphylaxis is the most severe form of an allergic/hypersensitivity reaction consisting of mucocutaneous, respiratory, and/or gastrointestinal involvement [1]. The global incidence of anaphylaxis is between 50 and 112 episodes per 100,000 person-years with the estimated lifetime prevalence being 0.3%-5.1% [1]. In this report, we discuss the case of a 38-year-old male presenting with an anaphylactic reaction to the placement of Surgicel following insertion of a permacath.

## Case presentation

A 38-year-old male with a past medical history of stage V chronic kidney disease (CKD) secondary to hypertensive nephropathy, hypertension, and normocytic anemia secondary to CKD presented to the Emergency Department for emergent dialysis and placement of a permacath at the direction of his Nephrologist. The patient was admitted and scheduled for surgery later that day due to acute CKD with worsening laboratory abnormalities. At the time of admission, the patient was working with a transplant team and had a kidney transplant scheduled in two months’ time. A pre-operative assessment was done, and the patient noted no known allergies to any medications, food, or environmental triggers. Surgery for permacath placement was completed without complication and the patient was sent to the medical floor for observation and to begin dialysis. Upon routine assessment, the patient’s dressing was noted to be saturated with blood, and Surgicel was placed at the site to achieve hemostasis. Within 5-10 minutes of placing the Surgicel, the patient experienced sudden onset of diaphoresis, shortness of breath, nausea, and pruritis of the upper and lower extremities. The patient’s home medications were not started at this point in the patient's care. The Rapid Response team arrived, and per the team the patient was diaphoretic, vitals were noted to be tachycardic to 120s, blood pressure 114/74, and oxygen saturation was 92% on room air. On physical exam, the patient had wheezing in all lung fields and facial angioedema. Surgicel was removed by the surgical team at the bedside. The surgical wound site appeared clean with no signs of local infection, swelling, erythema, urticaria, or purulent drainage. He was then placed on 100% oxygen via a non-rebreather mask and administered Solumedrol 125mg intravenously. The patient was also given Benadryl 50mg intravenously and 0.3mg of Epinephrine intramuscularly, at which time intubation was discussed to protect his airway. The patient was immediately transferred to the ICU after administration of Epinephrine where symptoms began to improve, and intubation was not indicated. Per ICU team evaluation, the patient showed improvement in stridor and wheezing, although tongue swelling was still noted, and he was started on Solumedrol 60mg intravenously every eight hours for 24 hours with close monitoring for worsening airway compromise. After a few hours, the non-rebreather was removed, and oxygen saturation remained at 100% on room air. A few hours after removing the offending agent and completing treatment, the patient was stable and returned to the medical floors. Dialysis was initiated the following day. The patient was assessed the next day for signs and symptoms of similar reactions. The patient remained asymptomatic until discharge.

## Discussion

Surgicel absorbable hemostat is a sterile absorbable knitted fabric prepared by the controlled oxidation of regenerated cellulose. It is used to accelerate clotting intraoperatively and postoperatively, although the exact mechanism by which it does so is not fully understood. After being saturated with blood, it swells into a gelatinous mass, which aids in clot formation, serving as a hemostatic adjunct in the control of local hemorrhage [2]. Currently, in the SURGICEL Family Scientific Literature, the adverse reactions noted include a stenotic effect when applied as a wrap during vascular surgery, paralysis, and nerve damage when used in proximity to foramina in bone, and prolongation of drainage in cholecystectomies [2]. When applied on surface wounds, stinging has been reported [2].

Anaphylaxis is an acute, severe, and potentially life-threatening systemic hypersensitivity reaction. International consensus on anaphylaxis defines anaphylaxis as a serious reaction with a rapid onset that can be fatal [3]. It is often mediated by IgE and results in mast cell and basophil mediator release. Clinical symptoms may involve many organ systems including cardiovascular, respiratory, cutaneous, and gastrointestinal. Cutaneous symptoms include urticaria, angioedema, and generalized erythema, and itching. Respiratory symptoms may consist of dyspnea, bronchospasm, laryngeal edema, and swelling of the tongue. Cardiovascular involvement includes any significant change in heart rate, hypotension, arrhythmia, or acute coronary event. Gastrointestinal symptoms include nausea, vomiting, diarrhea, etc.

The diagnosis of anaphylaxis is highly likely if one of the three following criteria is met. The first of these criteria includes either (a) acute onset of mucocutaneous and respiratory symptoms such as pruritis, flushing, urticaria, angioedema, wheezing, stridor, hypoxemia or cyanosis or (b) hypotension or end organ damage such as encephalopathy or acute kidney injury. The second of these criteria includes the presentation of two or more of the following complications, including mucocutaneous symptoms, respiratory symptoms, hypotension, or end-organ hypoperfusion, immediately after exposure to a known allergen. Finally, the third of these criteria includes reduced blood pressure shortly after exposure to a known allergen [4]. Following Surgicel placement, the patient developed sudden, diffuse, pruritus, and facial angioedema, as well as dyspnea, wheezing, and oxygen saturation of 92%. Referring to the criteria mentioned above, mucocutaneous involvement and respiratory involvement were apparent, therefore confirming the diagnosis of anaphylaxis.

Airway management followed by decontamination of the offending agent is paramount. Following these precautions, intramuscular epinephrine should be given. Injectable epinephrine is universally accepted as first-line therapy for anaphylaxis and may counteract pathophysiological changes in anaphylaxis via alpha-1 adrenergic receptors to induce vasoconstriction, which prevents and diminishes tissue edema, hypotension, and distributive shock [5,6]. It also acts on beta-2 adrenergic receptors to dilate airways and further block the release of histamine by mast cells [5]. Histamine increases vascular permeability and vasodilation, which leads to tissue hypoperfusion [6]. H1 and H2 antihistamines may also be helpful in treating cutaneous manifestations of anaphylaxis.

Identification of the cause of anaphylaxis is essential to prevent future recurrence, and for the safety of the patient. Often, inciting agents involve food, drugs, and insects, but in up to 20% of events, the trigger is not identified [7]. Currently, there has only been one other published case of an allergic reaction to oxidized cellulose, a case report by Royds et al. [8]. The patient we are presenting had an anaphylactic reaction to Surgicel following placement of a permacath. With another case of an allergic reaction being reported, it is essential for medical providers to be aware of this potential reaction when determining which hemostatic agents should be used in practice.

## Conclusions

Surgicel is a hemostatic agent commonly used intraoperatively and postoperatively. Although its use is widespread and generally regarded as safe, it is essential to remain vigilant after its placement considering the documented reports of an allergic reaction, including the case presented in this report, of anaphylaxis. Since Surgicel-induced anaphylaxis is a rarely reported hypersensitivity reaction, it is important to raise awareness of such reaction when it occurs. Early recognition of the condition is essential for immediate treatment in order to improve patient outcomes.
